# Coping with the economic burden of Diabetes, TB and co-prevalence: evidence from Bishkek, Kyrgyzstan

**DOI:** 10.1186/s12913-016-1369-7

**Published:** 2016-04-05

**Authors:** Matthias Arnold, David Beran, Hassan Haghparast-Bidgoli, Neha Batura, Baktygul Akkazieva, Aida Abdraimova, Jolene Skordis-Worrall

**Affiliations:** Munich Center of Health Sciences, Ludwig-Maximilians-Universität, Munich, Germany; Institute of Health Economics and Health Care Management, Helmholtz Zentrum München, Neuherberg, Germany; Division of Tropical and Humanitarian Medicine, Geneva University Hospitals and University of Geneva, Geneva, Switzerland; Institute for Global Health, UCL, London, UK; Health Policy Analysis Centre, Bishkek, Kyrgyzstan

**Keywords:** Diabetes, Tuberculosis, Coping strategy, Burden of disease, Kyrgyzstan, Cost analysis, Co-infection, Co-prevalence

## Abstract

**Background:**

The increasing number of patients co-affected with Diabetes and TB may place individuals with low socio-economic status at particular risk of persistent poverty. Kyrgyz health sector reforms aim at reducing this burden, with the provision of essential health services free at the point of use through a State-Guaranteed Benefit Package (SGBP). However, despite a declining trend in out-of-pocket expenditure, there is still a considerable funding gap in the SGBP. Using data from Bishkek, Kyrgyzstan, this study aims to explore how households cope with the economic burden of Diabetes, TB and co-prevalence.

**Methods:**

This study uses cross-sectional data collected in 2010 from Diabetes and TB patients in Bishkek, Kyrgyzstan. Quantitative questionnaires were administered to 309 individuals capturing information on patients’ socioeconomic status and a range of coping strategies. Coarsened exact matching (CEM) is used to generate socio-economically balanced patient groups. Descriptive statistics and logistic regression are used for data analysis.

**Results:**

TB patients are much younger than Diabetes and co-affected patients. Old age affects not only the health of the patients, but also the patient’s socio-economic context. TB patients are more likely to be employed and to have higher incomes while Diabetes patients are more likely to be retired. Co-affected patients, despite being in the same age group as Diabetes patients, are less likely to receive pensions but often earn income in informal arrangements. Out-of-pocket (OOP) payments are higher for Diabetes care than for TB care. Diabetes patients cope with the economic burden by using social welfare support. TB patients are most often in a position to draw on income or savings. Co-affected patients are less likely to receive social welfare support than Diabetes patients. Catastrophic health spending is more likely in Diabetes and co-affected patients than in TB patients.

**Conclusions:**

This study shows that while OOP are moderate for TB affected patients, there are severe consequences for Diabetes affected patients. As a result of the underfunding of the SGBP, Diabetes and co-affected patients are challenged by OOP. Especially those who belong to lower socio-economic groups are challenged in coping with the economic burden.

**Electronic supplementary material:**

The online version of this article (doi:10.1186/s12913-016-1369-7) contains supplementary material, which is available to authorized users.

## Background

When confronted with long term illness, households face not only a physical and mental burden, but also an economic burden. The economic burden of illness commonly includes the direct costs of treatment, drugs, transport and fees and informal payments, and the indirect costs of a reduction in their ability to generate income [1]. High levels of health spending can exceed a household’s capacity to pay, sometimes resulting in the sale of assets or the incurring of debt. Health spending exceeding a certain threshold of a household’s ability to pay is referred to in the literature as catastrophic expenditure and this is frequently the precursor to the medical poverty trap [2]. There is substantial evidence that health systems financed with out of pocket payments (OOP), are more likely to cause catastrophic health expenditure and resulting poverty, particularly in low- and middle-income countries (LMICs) [2–4].

To meet the financial costs of ill health, households may use a range of coping strategies such as selling household assets, reallocating spending from consumption and investment, borrowing money or working extra hours [5–7]. For tuberculosis (TB) care, the most commonly reported strategies are borrowing money, selling assets, using savings and transfers from relatives [8]. While such strategies can help to cope with the immediate economic burden [9], they can also leave the household at risk of long-term poverty [10]. Coping strategies are often categorised into detrimental strategies i.e. borrowing money or selling livelihood assets, and non-detrimental strategies, i.e. income or savings, labour substitution or social networks [6, 11, 12]. Detrimental coping strategies draw on the households’ ability to generate future income or leave the household indebted and thus at higher risk of economic vulnerability and poverty in the long run [10, 11, 13–16]. Empirical research has shown that detrimental coping strategies are more likely to be used by households with lower income and higher health expenditure [11, 17].

TB is a major public health threat in many LMICs and its control and prevention are priorities for many national and international health authorities, and non-governmental organisations [18]. Diabetes and TB have strong co-morbidities, with both the incidence and severity of TB being affected by Diabetes [19–22]. The rising prevalence of Diabetes in many LMICs thus poses an increasing risk for the control and prevention of TB [23–25]. As such, while this case study is focused on a single city in a LMIC with a developing health care system, the findings may have relevance for other countries in the process of developing their health care system.

Kyrgyzstan is a former Soviet Republic in Central Asia. It is a lower-middle income country with a Gross National Income per capita of $880 in 2010 [26]. In Kyrgyzstan, the Diabetes prevalence rate among the population aged 20 to 79 years, is at 6.3 % [27] and TB prevalence was estimated at 190 per 100,000 in 2013 [28]. Kyrgyzstan is one of the 18 high-priority countries in the Stop TB Plan of the WHO region [29] and one of the 27 countries with high rates of multidrug-resistant tuberculosis (MDR-TB) [30].

TB medicine and treatment is based on Direct Observed Treatment Short course (DOTS) and DOTS plus programs for the treatment of MDR-TB. DOTS is available throughout Kyrgyzstan, but DOTS plus is limited to some areas of Kyrgyzstan. Medicines for DOTS and DOTS plus programs are supplied mainly through the Global Fund and government funds. Patients do not pay for TB medicines, but for other supporting medicines and treatments e.g. vitamins, which are not provided for free. Financial barriers may also exist with regards to paying for transportation for outpatient management of TB and from first to secondary care facility when patients are referred. While some local authorities do reimburse for transport cost, this is not the standard for all health facilities. Similar challenges were also found for people with Diabetes in a previous study by Abdraimova, Beran [31]. In addition, poor purchasing practices were found in the management of Diabetes, resulted in Diabetic patients needing to purchase some or all of their diagnostic and therapeutic appliances and medicines in private sector [32].

In recent years, Kyrgyzstan has undergone major structural reforms in the health sector, improving the provision of universal health care. In 1996, the national health care reform programme, called Manas, was adopted, which had the improvement of access to health care as one of its pillars [33]. In 2002, a State-Guaranteed Benefit Package (SGBP) was introduced providing a list of basic health services free at the point of use [33, 34]. The SGBP provides free primary care for the entire population and referral care against a flat co-payment for the insured population. With help of the SGBP, access to health care has significantly improved in the last years [33]. Jakab et al. [35] reported results of a survey in 2009 stating that the proportion of people needing but not seeking care dropped from 11 % in 2000 to 4 % in 2009. Despite the fact that insurance is mandatory in principle, a World Bank case study in 2013 found that about 30 % of the population remain uninsured in practice [34]. For the uninsured, the level of co-payments is almost twice as high as for the insured [34]. However, there are exemptions from co-payments for vulnerable populations, such as children under five, pensioners above 75 years of age, disabled people, etc. Certain medical conditions are also exempt from co-payments including diseases that require high use of health services or for infectious diseases. Type 1 and 2 Diabetes are exempt from co-payments due to the high required use of health services and TB is exempt due on its infectious nature [34]. While OOP have declined from 2001 to 2007, the remaining economic burden of health payments is still considered to be significant [33]. In addition to formal co-payments, a report in 2012 extrapolated that informal payments for meals, medicines, sundries and payments to health workers are made at inpatient level and estimated the funding gap of the SGBP to be at 34.8 % [36]. This underfunding suggests a high rise of OOP to meet the funding gap if providers are not able reclaim expenses from the state.

A households’ choice of strategy for coping with health expenditure can have a sizeable impact on the creation or prevention of catastrophic health spending [1]. Identifying patterns in coping strategies and determinants of the selection of strategies can help both in understanding the financial context of affected households and tailoring social protection mechanisms accordingly. In Kyrgyzstan, research on coping strategies with OOP is not yet available and their impact on financial protection, while there is evidence that the funding gap of the SGBP requires ongoing reliance on OOP. Focusing on TB, Diabetes and co-affected patients allows us to compare variations in the economic burden of care seeking, and the choice of coping strategies to bridge state funding gaps. This paper aims to explore the economic burden and financial coping strategies of households affected by Diabetes, TB and co-prevalence. Our study adds to evidence of the effects of recent political reforms in Kyrgyzstan and contributes to existing research on households’ strategies of coping with economic burden by introducing the double burden of two merging diseases in a lower-middle income country.

## Methods

### Study design and data

This study uses data collected from a cross-sectional survey administered in 2010 as part of a larger study combining quantitative patient information and qualitative caregiver information to understand and improve the management of Diabetes and TB in Bishkek, Kyrgyzstan. Patient data was collected using an adapted questionnaire originally used to collect data on TB care seeking in Cape Town, South Africa [37]. The questionnaire was translated from English to Russian and then back-translated to ensure accuracy and coherence. The english version of the questionnaire can be found in Additional file 1. Exit interviews were conducted with 309 adult patients, and administered by experienced local health systems researchers at four health facilities which were most likely to provide care for Diabetes and TB patients, the primary and secondary care facilities for Bishkek (City Endocrinology Dispensary and the City TB Hospital) and the tertiary facilities, which receive referred patients from all of Kyrgyzstan (the Endocrinology Department of the National Hospital and the National TB Centre). Diabetes patients are interviewed in dispensaries and hospitals to include both outpatient and inpatient care, while TB patients are interviewed in the two hospitals which are most frequented. Ethical clearance was obtained from University College London (Project 0025/001). This data collection was part of the project “Diabetics in Kyrgyzstan”, which was also approved locally by the Committee on bioethics under the Ministry of Health of Kyrgyzstan. All adult patients in the waiting room on a given day were invited to participate in the survey after receiving their treatment and giving written informed consent. Interviews were on consecutive days, starting from 3 September 2010 and continuing until the sample size was achieved on 1 November 2010. Each interview lasted approximately one hour. A simple quota was used, resulting in a final sample of 138 patients with Diabetes (either Type 1 or Type 2), 139 patients with TB, and 32 patients with both illnesses.

The survey collected data on health expenditure (such as direct medical and non-medical costs, formal and informal treatment costs), socio-economic status (such as age, gender, education level, employment status, and household size) and financial coping strategies of patients (income and savings, social welfare and donations, support from social networks, borrowing money or selling household assets). Education was measured in levels of completed schooling (primary, secondary, tertiary). Employment status was categorised as: being out of work (unemployed or not able to work due to health issues), working in an informal arrangement (subsistence farming, self-employment or housekeeping), being formally employed (public or private sector) or retired (in retirement or pensioner).

The patient survey included specific information about household income and health expenditure in the national currency, Kyrgyz Som (KGS). The exchange rate was 46.14 KGS to 1.00 USD in 2011 [38]. Health expenditure included three components: the cost of travel to the health facility, informal payments made during the hospital visit, and all formal payments incurred during the visit including medicines, diagnostic tests, doctors’ fees, etc. Data were collected as aggregates for these components for the most recent overnight admission, observation or emergency room visit and outpatient visit. Patients were also asked for the reason for visiting to select only those visits related to Diabetes and TB. These cost components were then multiplied by the number of reported visits in the last 90 days, to get an estimate of health expenditure over this time span, while reducing the risk of recall bias. Health facility visits are compared for both single diseases and the co-prevalence.

Capacity to pay (CTP) was approximated by effective income [2, 39]. Effective income represents household income after subsistence needs are met. In urban settings, such as Bishkek, this effective income is often used to calculate CTP [2, 40, 41]. To reflect economies of scale in households with more members, household income was transformed into equivalence income. This was done by dividing household income by household size to the power of 0.56. This equivalence scale had been empirically derived from a multi-country regression of household survey data from 59 countries [2, 40]. Minimum food expenditure was subtracted from equivalence income and the remainder used as a measure of CTP for health expenditure and a proxy for poverty risk [40]. The average minimum food expenditure was derived from a World Bank calculation based on the Kyrgyzstan Integrated Households survey 2010 [42].

A commonly used measure of catastrophic health expenditure is to estimate health expenditure proportional to CTP. When using the CTP approach, a threshold of 40 % is commonly used [2]. If health expenditure exceeds the threshold and more than 40 % of the household’s CTP is being spent on health, a catastrophic impact on the household may be expected [2]. The catastrophic payment method was chosen over the measurement of impoverishment. The impoverishment approach measures if patients drop below a poverty line after health spending. Measuring impoverishment is however dependent on an agreed poverty line. While a national poverty line is available in Kyrgyzstan, a World Bank report in 2011 [26] found that this national poverty line is not representative of the actual subsistence level.

The selection and definition of coping strategies, is adopted from the analytical framework developed by McIntyre et al. [7]. To better represent the post-communist context of Kyrgyzstan a coping strategy called ‘social welfare support and donations’ is added. Additionally, social networks, such as family and friends, are included in this analysis and represent informal social protection mechanisms, as described by Russell [1]. As such, the complete list of coping strategies included in this study is as follows: income or savings, social welfare support or donations from employers or agencies; support from social networks like friends and family; borrow money; and raise money by selling assets. Patients were asked if they used these strategies for coping with the health care spending experienced in their health care seeking in the last 90 days, but could also indicate that they had used other coping strategies not listed in the response options.

### Coarsened exact matching

Comparing catastrophic health spending and coping strategies within the three patient groups without controlling for confounding by differences in the socio-economic status can result in overestimation of the effect of the disease or co-prevalence. Matching the patient groups according to their different socio-economic statuses helps identify the unbiased effect of the disease on financial coping strategies and the occurrence of catastrophic health spending. Nonparametric matching, such as CEM, allows reducing these imbalances. While the regression models still need to control for the socio-economic imbalances, the model dependence is much lower than without matching [43]. Multilevel matching is a very common technique for the analysis of observational data and has been employed in many recent studies analysing the economic burden of disease [44–47]. Coarsened Exact Matching (CEM) was used here to account for the socio-economic differences in the patient cohorts.

CEM aims at finding exact matches, but interprets “exact” as identical within a coarsened range of similar covariates. CEM finds categories of similar covariates based on distributions or intuitive division and matches exactly within these categories [47, 48]. The detailed methodology behind CEM can be read elsewhere [49, 50]. CEM has advantages over other matching algorithms like propensity score matching and exact matching, because it allows for the matching of groups with unequal numbers of observations and allows a degree of imprecision in the matching [47, 51]. This allows matching even when observations are not precisely exact, as long as they can be coarsened into similar categories. While CEM was originally designed to match one treated group to a untreated control group, it allows matching of multi-level treatment [43, 49].

In this study, CEM was used to match observations in the three patient groups, Diabetes, TB and co-prevalence. CEM was used here to control confounding by differences in the socio-economic background of patients affected by the two diseases or co-affected. The matching variables were: 1) gender, 2) age in years, 3) education level, 4) employment status and 5) equivalence income to control for differences in the capacity to pay, adjusted for household size.

### Data analysis

Data were analysed using, version R 3.0.2. In the descriptive statistics, all mean calculations include 95 % confidence intervals. For variables with skewed distributions, such as income and expenditure, we include their medians. Item non-response was checked and found to be highest for household income (5.5 %) and employment status (5.1 %). Household income and employment were analysed for non-response bias in relation to other socio-economic variables such as education, age, household size and gender. Non-response bias could be ruled out and list-wise deletion was used in the analyses.

Regression models were used to analyse the association between socio-economic background and coping strategies. For each coping strategy, a logit model was conducted using the GLM function with a binomial logit link in R. The socio economic variables used in these models (as independent variables) were: gender, age, education, employment and equivalence income in tertiles^1^.

Odds ratios were calculated to compare the likelihood of spending money on health care, engaging in catastrophic health spending and coping strategies between the three patient groups. Three patient group variables were generated (TB vs Diabetes, Co-affected vs Diabetes, Co-affected vs TB) to allow pairwise subgroup comparison. The odds ratios were determined with regression models for binary categorical variables using generalised linear models with logistic distribution functions i.e. logistic regressions [52]. As pointed out earlier, matching only reduces the imbalance between the patient groups, but does not completely eliminate it. Thus the regression models should include all covariates of the original, unmatched model [43, 49]. The regression models thus control for the covariates: 1) gender, 2) age in years, 3) education level, 4) employment status and 5) equivalence income. All odds ratios were estimated for matched and unmatched observations to allow a comparison of the association of the patient groups alone and the imbalanced association of patient group plus socio-economic background. While the models of the unmatched regressions produce estimates of the combined association of disease and socio-economic status, the logistic regressions of the matched observations, shows associations of the disease alone. All regression models were tested for influential outliers using Bonferroni statistics and influence index plots from the R package CAR, version 2.0-25. From the same R package, variance inflation factors were used to test multi-collinearity in all regression models.

## Results

### Differences in patient profile

Table 1 presents summary statistics of the main variables included in the analysis. On average, Diabetes patients are 55 years old, while TB patients are 30 years old (*p* < 0.001). Co-affected patients are approximately the same age (54 years) as the Diabetes patients. While TB patients have an equal gender ratio, Diabetes patients are mostly female (70 %) and co-affected patients are mostly male (63 %). Almost all patients have completed secondary school. The only differences are in the number of patients with completed tertiary education; while 54 % of Diabetes patients and 61 % of co-affected patients have completed tertiary education, only 31 % of TB patients have the same level of education. Half of Diabetes patients are retired, while 61 % of TB patients are in informal or formal employment. While co-affected patients are in the age range of Diabetes patients, they are only half as likely to be retired, but more than twice as likely to earn money in informal arrangements. TB patients are younger and accordingly, more likely to be in paid work than Diabetes patients. Although co-affected patients are the same age as Diabetes patients, they do not use pensions as often. Instead of pensions, co-affected patients earn money in informal arrangements.

**Table 1:** Descriptive statistics by patient group

Variable	Description	All patients	Only Diabetes	Only TB	Co-affected
Observations	N	309	138	139	32
Age	Mean	43.67	54.68	30.28	53.91
	(95 % CI)	(41.71 : 45.64)	(52.46 : 56.90)	(28.14 : 32.43)	(50 : 57.82)
Gender (%)	Female	58.06	70.29	50.36	37.50
	Male	41.94	29.71	49.64	62.50
Education (%)	No schooling	0.99	1.45	0.72	0.00
	Primary	2.96	2.90	2.88	3.12
	Secondary	50.66	39.86	64.75	28.12
	Tertiary	45.39	54.35	30.22	62.5
Employment (%)	Unemployed	21.77	16.67	24.46	21.88
	Informal	30.61	19.57	35.97	40.62
	Formal	18.37	13.04	23.74	6.25
	Retired	29.25	50.00	5.76	28.12
Household size	Mean	3.84	3.40	4.13	4.41
	(95 % CI)	(3.61 : 4.07)	(3.03 : 3.77)	(3.81 : 4.45)	(3.73 : 5.08)
Household income in KGS	Median	4500	3841	5000	4000
	Mean	7559	6562	9190	5097
	(95 % CI)	(6208 : 8910)	(5258 : 7866)	(6455 : 11925)	(3467 : 6726)
Equivalence income in KGS^a^	Median	2199	2595	2702	1833
	Mean	3989	3683	4704	2393
	(95 % CI)	(3288 : 4691)	(3023 : 4343)	(3273 : 6135)	(1602 : 3184)
Total health expenditure in KGS	Median	22	126	0	0
	Mean	404	635	177	366
	(95 % CI)	(280 : 528)	(378 : 891)	(104 : 249)	(59 : 672)
as informal payments in hospitals in KGS	Median	0	0	0	0
	Mean	55 (13 %)	0 (0 %)	109 (61 %)	63 (17 %)
as travel cost in KGS	Median	0	0	0	0
	Mean	140 (36 %)	254 (40 %)	49 (27 %)	43 (11 %)
Health spending of CTP^b^ (%)	Median	1.19	6.18	0.00	3.12
	Mean	40.70	71.96	11.06	31.75
	(95 % CI)	(11: 70)	(10: 134)	(4: 18)	(-4 : 68)
Visits to collect medication in the last 90 days (N)	Mean	1.55	1.80	0.94	3.22
	(95 % CI)	(1.19 : 1.92)	(1.58 : 2.01)	(0.20 : 1.68)	(2.06 : 4.38)
Outpatient visits in the last 90 days (N)	Mean	2.28	3.16	1.07	3.81
	(95 % CI)	(2.03 : 2.54)	(2.77 : 3.55)	(0.86 : 1.27)	(2.73 : 4.89)
ER visits in the last 90 days (N)	Mean	0.15	0.11	0.17	0.19
	(95 % CI)	(0.09 : 0.20)	(0.02 : 0.21)	(0.09 : 0.24)	(-0.01 : 0.39)
Inpatient admissions in the last 90 days (N)	Mean	0.98	0.83	1.13	0.97
	(95 % CI)	(0.88 : 1.07)	(0.66 : 1.00)	(1.02 : 1.23)	(0.81 : 1.12)
Number of coping strategies	Mean	1.80	1.90	1.73	1.66
	95 % C.I.	(1.69 : 1.90)	(1.76 : 2.04)	(1.56 : 1.91)	(1.32 : 1.99)
by income or savings	N	208	77	106	24
by social welfare or donations	N	109	72	30	7
by social networks	N	173	78	77	18
by borrowing money	N	31	15	13	3
by selling assets	N	36	20	15	1

^a^KGS = Kyrgyz Som: US$ 1.00 = KGS 46.14 (at average 2011 exchange rate)

^b^Capacity to pay (CTP) is defined as equivalence income reduced by minimum food expenditure

Equivalence income is highest in TB patients with 4704 KGS (106 USD), but this is not significantly higher (*p* = 0.183) than in Diabetes patients with 3683 KGS (83 USD). Co-affected patients have the lowest equivalence income with 2393 KGS (52 USD) and are significantly poorer than Diabetes (*p* = 0.008) and TB patients (*p* = 0.004).

**Fig. 1:**
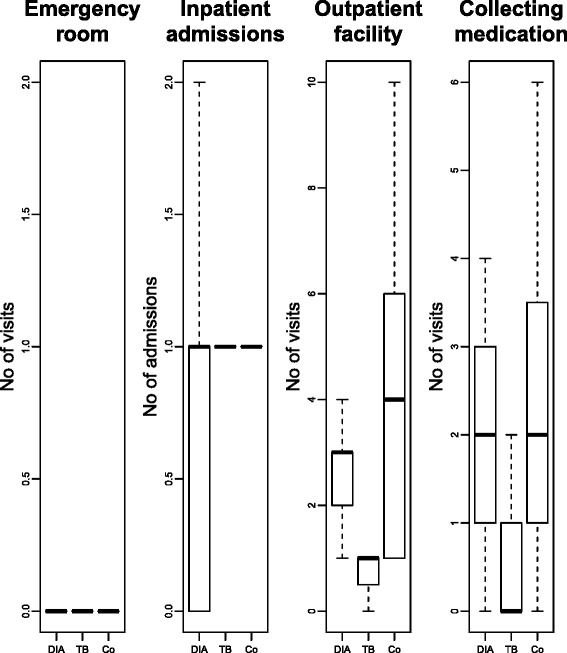
Box plot for health facility visits, matched observations

Figure 1 illustrates the differences in number of visits in matched observations. The mean number of inpatient admissions is around 1 and mean visits to the emergency room are around 0 for all patient groups. There are, however, differences in the number of outpatient visits and visits to collect medication. Diabetes and co-affected patients have a mean of 3 and 4 visits to outpatient facilities respectively, while TB patients only visit the outpatient facility once a month. Diabetes and co-affected patients need on average 2 visits per month to collect medication, while TB patients have on average 0 visits to collect medication. Significance tests prove that the differences between TB and Diabetes patients, as well as the differences between TB and co-affected patients, are significant. There is no significant difference between Diabetes and co-affected patients. In outpatient and medication collection visits, it becomes apparent that the co-affected patients face the double burden of Diabetes care and TB care, with the number of visits being exactly the sum of the other single disease patient groups.

The analysis of health expenditure in Table 1 shows that health expenditure is highest for Diabetes patients and lowest for TB patients. The medians in Table 1, illustrate however, that means comparison might not be appropriate, since there is a substantial number of patients who did not face any health expenditure in the last 90 days, despite using health services. From Table 5, it can be seen that Diabetes patients are significantly more likely to spend money for health care than TB patients and this finding is consistent after matching the observations for socio-economic status. Co-affected patients are in the middle between the single disease groups with a higher likelihood to face OOP than TB patients, but a smaller likelihood than Diabetes patients. Informal payments in hospitals and travel cost to total health expenditure can further be distinguished from co-payments for medication due to underfunding in the SGBP. Informal payments for outpatient care were not collected, which clearly limits this study with respect to the analysis of informal payments. In hospital settings, this study shows that all patient groups have median spending for informal payments of zero. Only few participants, all among TB or co-affected patients, reported to have informal payments. Similarly, travel spending was reported zero by most of participants, but for some patients getting to the health facility can be costly. This is more often the case for Diabetes patients with a mean spending of 254 KGS. TB and co-affected patients have mean spending of 49 KGS and 43 KGS. The remaining health spending can be seen as co-payments within the SGBP for medicines, diagnostic tests, consultation and others.

### Differences in coping strategy users

After demonstrating the higher economic burden for Diabetes and co-affected patients, the focus of this section of analysis is on the financial strategies for coping with the economic burden of care. Table 2 shows the number of coping strategies a patient or her/his household uses and the percentages of patients using a specific strategy for their last care seeking event. 208 patients used income or savings, 173 use social welfare and donations, 109 use support from social networks, 36 had to sell household assets and 31 borrowed money. Among the 208 households using income or savings, 34 % use this as their only coping strategy. Among the 173 households using social networks, 23 % use this strategy as their only financing source. Table 3 shows that social network is used most of all strategies as an additional funding source. Social welfare or donations are rarely used as a single strategy. Borrowing money, selling assets, donations and social welfare are also much more likely to be used in combination with other strategies. Borrowing money is combined most often with at least two or three additional strategies.

**Table 2:** Coping strategies by number of strategies used simultaneously

Number of coping strategies used	All observations		Income or savings		Social welfare or donations		Social networks		Borrowing money		Selling assets	
0	12	4 %	-	-	-	-	-	-	-	-	-	-
1	122	40 %	71	34 %	10	9 %	39	23 %	0	0 %	3	8 %
2	109	35 %	82	39 %	44	40 %	72	42 %	5	16 %	15	42 %
3	50	16 %	41	20 %	40	37 %	46	27 %	10	32 %	13	36 %
4	14	5 %	12	6 %	13	12 %	14	8 %	14	45 %	3	8 %
5	2	1 %	2	1 %	2	2 %	2	1 %	2	6 %	2	6 %
total	309	100 %	208	100 %	109	100 %	173	100 %	31	100 %	36	100 %

**Table 3:** Combinations of coping strategies, by number of patients using at least two strategies, with patient group

Number of patients using at least two strategies	Income or savings (Dia,TB,Co)	Social welfare or donations (Dia,TB,Co)	Social networks (Dia,TB,Co)	Borrowing money (Dia,TB,Co)	Selling assets (Dia,TB,Co)
Income or savings		70 (39,24,7)	98 (30,57,11)	20 (7,11,2)	20 (7,12,1)
Social welfare or donations	70 (39,24,7)		71 (38,28,5)	20 (8,10,2)	10 (8,2,0)
Social networks	98 (30,57,11)	71 (38,28,5)		26 (12,11,3)	19 (11,8,0)
Borrowing money	20 (7,11,2)	20 (8,10,2)	26 (12,11,3)		9 (6,3,0)
Selling assets	20 (7,12,1)	10 (8,2,0)	19 (11,8,0)	9 (6,3,0)	

Additional regression analyses show that the socio-economic background influences the decision which a patient makes regarding what coping strategy to use. Five multivariate regression models were conducted to investigate association with socio-economic background variables for each of the five coping strategies. Table 4 shows significant association between employment status, age and equivalence income. Knowing that the socio-economic background varies within the three patient groups and knowing that socio-economic background is also associated with coping, raise this question whether choice of coping strategy is associated with disease or with socio-economic context of the patient arises. The next section compares coping in patient groups with balanced and imbalanced socio-economics and thus allows differentiating the association between patient groups and coping strategy.

**Table 4:** Regression results, associations between coping strategy and variables of socioeconomic background

	Income & savings	Social Welfare & Donations	Social Networks	Borrowing money	Selling assets
	Coeff. (S.E.)	Coeff. (S.E.)	Coeff. (S.E.)	Coeff. (S.E.)	Coeff. (S.E.)
(Intercept)	16.69	-1.16	15.22	-15.13	-16.48
	(994.52)	(1.51)	(992.70)	(1018.94)	(1006.01)
Age: for each year	-0.03 **	-0.00	0.01	-0.00	0.03 *
	(0.01)	(0.01)	(0.01)	(0.02)	(0.02)
Gender: female vs male	-0.47	0.10	0.43	-0.01	0.31
	(0.31)	(0.31)	(0.27)	(0.45)	(0.44)
Education: compl. primary vs no schooling	-14.85	1.85	-12.97	16.47	14.58
	(994.52)	(1.73)	(992.71)	(1018.94)	(1006.01)
Education: compl. secondary vs no schooling	-15.30	0.30	-14.58	13.85	13.60
	(994.52)	(1.45)	(992.70)	(1018.94)	(1006.01)
Education: compl. tertiary vs no schooling	-15.31	-0.27	-15.04	14.11	12.39
	(994.52)	(1.45)	(992.70)	(1018.94)	(1006.01)
Employment: informal vs unemployed	1.20 ***	-1.05 **	-1.01 ***	-1.92 ***	-0.23
	(0.41)	(0.43)	(0.38)	(0.70)	(0.70)
Employment: formal vs unemployed	1.01 **	-0.66	-1.30 ***	-0.73	0.70
	(0.46)	(0.45)	(0.43)	(0.60)	(0.62)
Employment: retired vs unemployed	0.50	1.37 ***	-1.08 **	-0.52	-0.33
	(0.46)	(0.48)	(0.46)	(0.65)	(0.71)
Equivalence income: second tertile vs first tertile	0.16	0.40	-0.16	-0.66	0.60
	(0.34)	(0.37)	(0.32)	(0.57)	(0.47)
Equivalence income: third tertile vs first tertile	0.41	0.93 *	0.25	-0.05	-0.23
	(0.36)	(0.37)	(0.33)	(0.50)	(0.55)
AIC	323.48	312.77	364.85	184.11	200.28
BIC	362.85	352.23	404.36	223.57	239.74
Log Likelihood	-150.74	-145.39	-171.43	-81.06	-89.14
Deviance	301.48	290.77	342.85	162.11	178.28
Num. obs.	265	267	268	267	267

^***^ *p* < 0.01, ^**^ *p* < 0.05, ^*^ *p* <0.1

### Catastrophic health expenditure and coping strategies

Using CEM, 175 of 310 observations could be matched, reducing the imbalance score of L1 by 88 %. Table 5 shows the odds ratios for matched and unmatched observations for three patient groups. While the models of the unmatched regressions produce estimates of the combined or imbalanced association of disease and socio-economic status, the logistic regressions of the matched observations, find the association with the disease alone. The odds ratio in the unmatched observations show four significant results: 1) TB patients are more likely to use income or savings than Diabetes patients, 2) Diabetes patients are more likely to use social welfare or donations than TB patients, 3) co-affected patients are less likely to use social welfare or donations than Diabetes patients and 4) co-affected patients are more likely to face catastrophic health spending than TB patients. When using the CEM approach to match the observations for socio-economic status, the logistic regression models show three significant results: 1) TB patients are less likely to engage in catastrophic health spending than Diabetes patients, 2) Diabetes patients are more likely to use social welfare or donations than TB patients and 3) co-affected patients are less likely to use social welfare or donations than Diabetes patients.

**Table 5:** Odds ratio of spending money for health care, engaging in catastrophic health spending and financial coping, matched and unmatched

	Unmatched (*n* = 310)			Matched (*n* = 175)		
	TB vs Diabetes	Co-affected vs Diabetes	Co-affected vs TB	TB vs Diabetes	Co-affected vs Diabetes	Co-affected vs TB
	OR (S.E.)	OR (S.E.)	OR (S.E.)	OR (S.E.)	OR (S.E.)	OR (S.E.)
Spending money on health care	0.41 (1.39)***	0.61 (1.60)	1.24 (1.65)	0.33 (1.54)**	0.48 (1.77)	1.18 (1.74)
Catastrophic health spending	0.65 (1.41)	1.68 (2.04)	3.82 (2.01)*	0.24 (2.12)*	2.03 (2.80)	3.44 (3.13)
Coping Strategies:						
Income or savings	2.91 (1.43)***	1.30 (1.70)	0.77 (1.83)	1.92 (1.57)	1.15 (1.84)	0.91 (1.85)
Social welfare or donations	0.42 (1.43)**	0.19 (1.85)***	1.97 (2.11)	0.18 (1.75)***	0.26 (2.05)*	3.26 (2.44)
Social Networks	0.88 (1.39)	1.19 (1.62)	0.93 (1.73)	1.16 (1.51)	0.98 (1.73)	1.03 (1.79)
Borrowing money	0.90 (1.67)	0.75 (2.09)	3.09 (2.65)	0.41 (2.62)	1.07 (2.54)	6.04 (3.60)
Selling assets	0.53 (1.62)	0.29 (3.00)	0.28 (3.21)	0.60 (1.88)	0.24 (3.13)	0.25 (3.35)

*** *p* < 0.01, ** *p* < 0.05, * *p* < 0.1

## Discussion

Diabetes and TB affect patients with different socio-economic and demographic characteristics. In this study, Diabetes patients are older and more likely to be female. They are more likely to have completed higher levels of education and to be pensioners. TB patients on the other hand are younger and have completed fewer levels of education. They are more likely to work, but as likely female as male. Co-affected patients are similar to Diabetes patients in age and education, but are more likely to be male and are less likely to live off pension income but more likely to earn money in informal arrangements. This is similar to case studies by Deshmukh, Shaw [53] and Pérez-Guzmán et al. [54] finding male dominance in co-affected patients. Additionally, co-affected patients have significantly lower equivalence income, due to lower household income in bigger households. Accordingly, co-infection affects Diabetes patients with low socio-economic status. This is supported by other studies identifying socio-economic factors as main drivers of TB infections [55–58].

The management of Diabetes and Tuberculosis in Kyrgyzstan is organised in vertical systems [59]. Vertical systems carry the risk of multiplying the burden of care-seeking experienced by patients with more than one disorder [60]. This was one of the reasons why the WHO and the International Union Against Tuberculosis and Lung Diseases advised coordinated management of the diseases in their collaborative framework [55, 60]. TB care receives support from international donors [61], while Diabetes care is financed solely through national budgets. In the face of the high rates of MDR-TB in Kyrgyzstan and the additional funding required to meet these challenges [62], government commitment to increase the SGBP funding is crucial. The call for more funding is even more urgent knowing that OOP still play an important role, especially for Diabetes care.

In this study, we find that the financial burden of care seeking is significantly greater in patients affected by Diabetes than TB and significantly greater for co-affected patients than TB patients due to their lower socio-economic status. The burden thus is regressive for these co-affected patients. As Skordis-Worrall et al. [63] point out, in Kyrgyzstan TB care is provided on inpatient basis, usually an admission for two to three months, followed by outpatient treatment for four months. Diabetes is mainly treated at outpatient level, but can also include inpatient admission. Diabetes care creates a bigger economic burden on patients and their households than TB care by demanding frequent outpatient visits, visits to collect medication and by the fact that insulin that should be free under the SGBP is often not unavailable and needs to be purchased from private pharmacies. The economic burden of co-prevalence is then especially high, since Diabetes and TB care are not offered in the same facilities, but require patients to visit both service providers. Since both informal payments and travel cost were only reported by few patients, therefore, it can be concluded that the main driver of the economic burden are payments for health facility visits and payments for unavailable medication.

As mentioned, TB is treated in inpatient care in Kyrgyzstan in the urban setting of Bishkek, while Diabetes care demands frequent visits to outpatient facilities or dispensaries to collect medication. This can affect the measurement of the economic burden of TB care in Kyrgyzstan compared to other countries where TB care is provided in communities. One can speculate that hospital-based care is likely to have smaller formal, direct costs, but higher indirect and opportunity cost for patients than community-based care. Considering this, one would expect that informal and travel cost to be higher for TB patients. While there are some TB patients who experience high informal cost, this is not true for every patient. Khan et al. [64] found that in Pakistan, community-based care has smaller opportunity cost than hospital-based care and overall smaller patient cost. This finding was also supported by Okello et al. [65] in Uganda and Sinanovic et al. [66] in South Africa who found that rural community-based TB care is more cost-effective than hospital-based care and the overall economic burden for patients is smaller. Scale up of outpatient based DOTS programmes all over the country could be a method to reduce the economic burden for TB and co-affected patients.

The financing strategies used for coping with the economic burden can indicate how well the social protection system in Kyrgyzstan functions and whether adjustments are necessary. The primary income source is used dominantly as the main financial coping strategy. Social network support is often used as secondary or additional financial source. The primary income source is most often work income for TB patients, because of their relatively young age, and pensions for Diabetes patients. Co-affected patients despite being the same age as Diabetes patients, use pensions less often. When socio-economic differences are balanced between Diabetes and TB patients, TB patients are significantly less likely to experience catastrophic health spending. If unmatched observations are used, the association is however not significant. One possible interpretation is that the socio-economic context of Diabetes patients actually protects them better than TB patients. The main difference in the socio-economic background naturally is the older age and the access to social welfare support, such as pensions, which provide income even in time of illness. The matching also shows that the association between co-prevalence and catastrophic health spending is due to the differences in socio-economic context. Co-affected patients are much more likely to experience catastrophic health spending than TB patients, who are younger and from a higher socio-economic background. When those socio-economic imbalances are controlled for by matching, the association loses its significance.

Coping with the health burden poses a range of challenges and not all households have the capacity to avoid catastrophic health expenditure. These data have shown that, in this context, households in the three patient groups have different socio-economic profiles and their choice of coping strategy depends on their background. Reliable sources of money, such as income from salaries and pensions are used very frequently, but are often topped up with assistance from friends or family. These social networks also play an important role as a social protection mechanism in the absence of other income sources. This finding supports other studies finding that informal loans from family, friends or employer [67, 68] are very important for health financing. Coping strategies that draw on the household’s future income or capital stock are not used frequently in this context. While other studies found that loans from money lenders [14, 17] play a very important role in the financing of health expenditure and the dependency on loans increases with decreasing wealth [11], here borrowing money is used only rarely and is linked to unemployment rather than low income.

## Limitations

This study has a number of limitations which should be acknowledged. Firstly, there is no control group in the sample, the level of co-payments for other diseases is accordingly not available. It could be possible that the co-payments experienced by Diabetes or TB patients are thus not significantly different than in the general population.

Secondly, this study did not include some confounding variables, such as HIV status, if Diabetes was Type 1 or Type 2 or MDR-TB. HIV is, however, very rare in Kyrgyzstan with a prevalence of under one percent [69]; Diabetes patients were all adults and had an average age which speaks more for Type 2 than for Type 1 diabetes.

Thirdly, the small sample size (in particular co-affected patients) limits this study’s power in the analysis of the co-affected patient group.

Fourthly, the extent of economic burden and catastrophic health spending might be underestimated in comparison to the general population, since patient interviews were held at health facilities. Patients who chose to cope with the economic burden by not seeking treatment are thus excluded in this study sample. In addition, it should be noted that service provision should be free for Diabetes and TB patients under the SGBP and it is possible that the participant in this study might have not shared all information in the exit interview in the waiting rooms. It cannot be ruled out that not all information about health care spending was reported. This could mean that the true economic burden and the level of catastrophic health spending are underestimated.

Lastly, recent research on the capacity to pay methodology suggested using a larger basket of consumption good than food expenditure to reflect household basic consumption in a more realistic way. In absence of a new estimation of subsistence spending, the World Bank estimate based on food expenditure was used. However, capacity to pay may further be overestimated in this study and thus catastrophic health spending can be further underestimated [70].

## Conclusion

The health sector in Kyrgyzstan aims to offer treatment for Diabetes and TB free at the point of use, through the SGBP. While studies have shown that the implementation of the SGBP has improved access to health services and equity in care by protecting patients from some portion of the financial burden [33, 71], other studies have challenged this finding and claimed that OOPs are still existent and continue to impose a significant economic burden on affected households [34, 36].

This study provides evidence that Diabetes and co-affected households face significant financial burden. Due to the chronic nature of Diabetes, this economic burden has long-term implications for the economic survival of households. They do not appear to be fully protected from that burden by provisions from the State. As such, in order to continue reducing the dependence on OOP, and the economic burden of ill health faced by affected households, the funding gap in the SGBP needs closer attention. This study shows that OOP are greater for Diabetes affected patients and co-affected patients than for TB patients. While a previous study [36] attributed the funding gap in majority to informal payments in hospitals, these payments were not found to be very common in this study. This leads to the conclusion that the funding gap in the SGBP and the resulting co-payments for medication, tests and other supplies impose the economic burden on households. The funding of the SGBP needs to be improved in order to provide access to continuous free-of-charge health care and continue reducing the OOP, especially for Diabetes patients with low socio-economic status, outpatient care and lower socio-economic households in general.

In the urban setting of Bishkek, pensions play an important role in coping with the economic burden of Diabetes. For co-affected patients however, pensions are not sufficient or available and thus additional income generation in the informal sector is necessary. Co-affected patients are not only challenged by their disease and socio-economic status, but also by the structure of service provision. Since TB and Diabetes services are not being offered in the same facility, co-affected patients necessarily face the double burden. Scaling up the DOTS and DOTS plus programme to outpatient services within the Diabetes dispensaries can help reducing the burden.

## Additional file

Additional file 1: Patient Questionnaire. (DOC 427 kb)

## Abbreviations

CEM: coarsened exact matching
CTP: capacity to pay
KGS: Kyrgyz Som (currency)
LMIC: low and middle income country
MDR-TB: multidrug-resistant tuberculosis
OOP: out-of-pocket
TB: tuberculosis
USD: United States Dollar (currency)
SGBP: State-Guaranteed Benefit Package

## Footnotes

1 Equivalence income tertiles were from 0 to 1,680 KGS, from 1,680 to 3,390 KGS and from 3,390 to 27,000 KGS.
