# Supplementary material for: Coping with the economic burden of Diabetes, TB and co-prevalence: evidence from Bishkek, Kyrgyzstan
Source: BMC Health Serv Res. 2016 Apr 5;16:118. doi: 10.1186/s12913-016-1369-7 (PMC4822315; doi:10.1186/s12913-016-1369-7)
Supplement: Additional file 1: — Patient Questionnaire. (DOC 427 kb) [file 12913_2016_1369_MOESM1_ESM.doc]

| **DIABETES AND TUBERCULOSIS IMPACT STUDY**  A Collaboration between the Health Protection Agency of Kyrgyzstan and University College London | | | | | | | | | | | |
| --- | --- | --- | --- | --- | --- | --- | --- | --- | --- | --- | --- |
| **INTERVIEWER PLEASE READ OUT:**  Dear Sir/Madam,  We are trying to understand how illness affects different households. We would like to ask you a few questions about your experience of illness and how it has affected you and your family. This should not take more than 45 minutes. The information collected is confidential and the name and address of the respondent will not be used for any other purpose than this study. Names will not be linked to the information gathered.  You may refuse to answer any question and we can stop at any time. If you agree to participate please will you sign below.  Thank you.  **I agree to participate in this study: ………………………………………………………………** | | | | | | | | | | | |
| **Interviewer Name:** |  | | | | | | | | **Interviewee Name:** | |  |
| **Survey ID Number[[1]](#footnote-2):** |  |  |  |  |  |  |  |  | **Contact telephone number:** |  | |
| **Interview Date** | _____/_______/2010  dd mm | | | | | | | | **Physical address:** | |  |

1. **DEMOGRAPHIC CHARACTERISTICS**

| **Question** | | **Codes** | **Response** |
| --- | --- | --- | --- |
| **1.1** | What is your gender? | 1 = Male  2 = Female  (*Interviewer: you may just observe this*) |  |
| **1.2** | How old were you in years at your last birthday?  ***FILL IN THE NUMBER IN YEARS.***  ***DISCONTINUE IF UNDER 18*** | |  |
| **1.3** | What is the highest grade you completed in school or post school? | 1 = No Schooling  2 = Completed primary school  3 = Completed secondary school  4 = Completed post school qualification  99 = Refused |  |
| **1.4** | Which of the following statements best described your work situation?  ***MULTIPLE MENTION POSSIBLE*** | 1 = I work only around the home (childcare, cooking, cleaning, taking care of livestock etc)  2 = I am a small scale farmer  3 = I am self employed but not as a farmer  4 = I am in formal employment (for salary or wages from a single employer) in the public sector  5 = I am in formal employment in the private sector (includes NGOs)  6 = I am not able to work due to my health condition  7 = I am not working but I am **looking** for work  8 = I am not working but I am **not looking** for work  9 = I am retired or a pensioner  10 = Don’t know  99 = Refused |  |
| **1.5** | How many people normally eat together where you live? | *(fill in the number)* |  |
| **1.6** | Taking those who eat with you as your household. Does your household own any of the following items?  ***MULTIPLE MENTION POSSIBLE*** | 1 = Car  2 = Motorcycle  3 = Bicycle  4 = Refrigerator  5 = TV set  6 = Computer  7 = Fixed line telephone  8 = Mobile/cellular phone  9 = Microwave  10 = Electric cooker  11 = House connected with electricity  12 = Piped water  13 = Flush toilet  14 = Stone/brick walls  15 = Tiled roof  99 = Refused |  |
| **1.7** | Considering all the work activities that the members of this household engage in. How much money does this household receive in a typical month from everybody (take home pay) including all earnings, pensions, grants, remittances, gifts of money or earnings from the sale of home grown/made products? ***FILL IN THE NUMBER*** | |  |

1. **PERSONAL HEALTH STATUS (SF8)**

| **Question** | | **Codes** | **Response** |
| --- | --- | --- | --- |
| **2.1** | Overall, how would you rate your health during the **past month**? | 1 = Excellent  2 = Very good  3 = Good  4 = Fair  5 = Poor  6 = Very poor |  |
| **2.2** | During the **past month**, how much did physical health problems limit your usual physical activities (such as walking or climbing stairs)? | 1 = Not at all  2 = Very little  3 = Somewhat  4 = Quite a lot  5 = Could not do physical activities |  |
| **2.3** | During the **past month**, how much difficulty did you have doing your daily work, both at home and away from home, because of your physical health? | 1 = None at all/  2 = A little bit  3 = Some  4 = Quite a lot  5 = Could not do daily work |  |
| **2.4** | How much bodily pain have you had during the **past month**? | 1 = None  2 = Very mild  3 = Mild  4 = Moderate  5 = Severe  6 = Very severe |  |
| **2.5** | During the **past month**, how much energy did you have? | 1 = Very Much  2 = Quite a lot  3 = Some  4 = A little  5 = None |  |
| **2.6** | During the **past month**, how much did your physical health or emotional problems limit your usual social activities with family or friends? | 1 = Not at all  2 = Very little  3 = Somewhat  4 = Quite a lot  5 = Could not do social activities |  |
| **2.7** | During the **past month**, how much have you been bothered by **emotional problems** (such as feeling anxious, depressed or irritable)? | 1 = Not at all  2 = Slightly  3 = Moderately  4 = Quite a lot  5 = Extremely |  |
| **2.8** | During the **past month**, how much did personal or emotional problems keep you from doing your usual work, school or other daily activities? | 1 = Not at all  2 = Very little  3 = Somewhat  4 = Quite a lot  5 = Could not do daily activities |  |

1. **USE OF MEDICAL SERVICES**

| **Question** | | **Codes** | **Response** |
| --- | --- | --- | --- |
| **3.1** | I now want to ask you about your use of health services over the last 3 months or 90 days. Please start by thinking of an event that happened about 3 months ago. Most of my questions will be about health services that you have used since that time. Can you remember something that you did or that happened to you about 3 months (90 days) ago?  ***RECORD:*** ……………………………………………………………………………………………………..……… | | |
| **3.2 ** | During the last 90 days, did you ever visit a HOSPITAL of any kind to get an kind of medical care for yourself, including just seeing a doctor or nurse just to get medication? | 1 = Yes  2 = No  88 = Don’t know  ***SKIP TO SECTION 3***  99 = Refused  ***IF NO, MAKE SURE NO HOSPITAL VISIT OF ANY KIND*** |  |
| **3.3** | During the last 90 days, how many times did you go to a hospital and end up spending at least 24 hours in a bed or stretcher?  ***ENTER NUMBER OF TIMES (88 = DON’T KNOW)*** | |  |
| **3.4** | During the last 90 days, how many times did you go to a hospital and end up spending LESS THAN 24 hours in a bed or stretcher, for example in an observation room, emergency room or casualty room?  ***ENTER NUMBER OF TIMES (88 = DON’T KNOW)*** | |  |
| **3.5** | During the last 90 days, how many times did you go to a hospital to see a doctor or a nurse as an outpatient (not an emergency room visit)?  ***ENTER NUMBER OF TIMES (88 = DON’T KNOW)*** | |  |
| **3.6** | During the last 90 days, how many times did you go to a hospital to get medications?  ***ENTER NUMBER OF TIMES (88 = DON’T KNOW)*** | |  |
| **3.7** | Before the times you visited a hospital during the last 90 days, how many OTHER times during the last YEAR did you spend at least one night in a hospital?  ***ENTER NUMBER OF TIMES (88 = DON’T KNOW)*** | |  |
| **3.8** | Now I would like to ask about what happened during some of these visits to the hospitals. Let’s start with you most recent hospital admission, observation room admission and outpatient visit. What was the name of the hospital where you had your most recent overnight admission?  ***RECORD:*** ……………………………………………………………………………………………………..………  ***(WRITE 00 IF NO OVERNIGHT ADMISSION)*** | | |
| **3.9** | What was the name of the hospital where you had your most recent observation room visit?  ***RECORD:*** ……………………………………………………………………………………………………..………  ***(WRITE 00 IF NO OVERNIGHT ADMISSION)*** | | |
| **3.10** | What was the name of the hospital where you had your most recent routine outpatient visit?  ***RECORD:*** ……………………………………………………………………………………………………..………  ***(WRITE 00 IF NO OVERNIGHT ADMISSION)*** | | |

| **Question** | | **Codes** | **Responses** | | | |
| --- | --- | --- | --- | --- | --- | --- |
|  | | | **Recent overnight admission** | | **Recent observation room/ emergency room visit** | **Most recent routine outpatient visit** |
|  | Why did you choose this facility?  ***MULTIPLE MENTION***  ***IF ‘OTHER’ PLEASE RECORD RESPONSE VERBATIM*** | 1 = Clean  2 = Good reputation (heard from others)  3 = Personal experience  4 = Has good equipment/ the right equipment  5 = Will give a thorough examination  6 = It is a specialised provider  7 = More chance of seeing a doctor  8 = Staff is friendly  9 = Waiting times are shorter  10 = It is closer to home/ convenient for transport  11 = I trust this service provider  12 = They will receive more attention at this provider  13 = The staff is more knowledgeable/ better trained  14 = They will have more privacy with this provider  15 = This is a safer provider  16 = They will have the necessary drugs available  17 = Affordable  18 = It is where I was taken/told to go  19 = It is the only facility I know of  20 = it is the only facility I can reach  66 = Other |  | |  |  |
| **2.6** | What main problem caused you to go to the hospital this time?  ***SINGLE MENTION ONLY*** | 1 = Heart disease  2 = Stroke  3 = Kidney disease  4 = Eye disease  5 = Leg or foot ulcer  6 = Cancer  7 = Lung disease  8 = Trauma  9 = Diabetes  10 = Childbirth  11 = Tuberculosis  66 = Other  88 = Don’t know |  | |  |  |
| **Question** | | **Codes** | **Responses** | | | |
|  | | | **Most recent overnight admission** | **Most recent observation room/ emergency room visit** | | **Most recent routine outpatient visit** |
| **2.6** | What other problems caused you to go to the hospital this time?  ***MULTIPLE MENTION POSSIBLE*** | 1 = Heart disease  2 = Stroke  3 = Kidney disease  4 = Eye disease  5 = Leg or foot ulcer  6 = Cancer  7 = Lung disease  8 = Trauma  9 = Diabetes  10 = Childbirth  11 = Tuberculosis  66 = Other  88 = Don’t know |  |  | |  |
| **2.6** | ***IF ADMITTED FOR LESS THAN 24 HOURS:***  How many hours did you spend at the hospital?  ***ENTER AS A DECIMAL I.E. 1H30MINS = 1.5HOURS*** | 88 = Don’t know  99 = Refused |  |  | |  |
| **2.6** | ***IF ADMITTED OVERNIGHT:***  How many nights did you spend at the hospital?  ***ENTER NO OF NIGHTS*** | 00 = Did not stay overnight  88 = Don’t know  99 = Refused |  |  | |  |
| **2.6** | ***IF ADMITTED OVERNIGHT:***  How many nights did you spend in ICU (intensive care unit)?  ***ENTER NO OF NIGHTS*** | 00 = Did not stay overnight  88 = Don’t know  99 = Refused |  |  | |  |

| **Question** | | **Codes** | **Responses** | | |
| --- | --- | --- | --- | --- | --- |
|  | | | **Most recent overnight admission** | **Most recent observation room/ emergency room visit** | **Most recent routine outpatient visit** |
| **2.6** | Did a doctor see you or only a nurse? | 1 = Doctor  2 = Nurse  88 = Don’t know  99 = Refused |  |  |  |
| **2.6** | ***IF SAW A DOCTOR:***  How MANY specialist doctors or surgeons?  ***ENTER NO OF DOCTORS*** | 00 = Did not see a doctor  88 = Don’t know  99 = Refused |  |  |  |
| **2.6** | ***IF SAW A DOCTOR:***  How MANY primary care doctors or surgeons?  ***ENTER NO OF DOCTORS*** | 00 = Did not see a doctor  88 = Don’t know  99 = Refused |  |  |  |
| **2.6** | What tests or procedures where done? | 1 = Blood test  2 = Urine test  3 = x-ray or ultrasound  4 = major surgery  5 = minor surgery  88 = Don’t know  99 = Refused |  |  |  |
| **2.6** | Did you receive any pills, injections or infusions? | 1 = Pills  2 = Injections  3 = IV Drips  66 = Other  88 = Don’t know  99 = Refused |  |  |  |
| **2.6** | How long did it take to get to this hospital this time?  ***ENTER AS A DECIMAL*** | 88 = Don’t know  99 = Refused |  |  |  |

| **Question** | | **Codes** | **Responses** | | |
| --- | --- | --- | --- | --- | --- |
|  | | | **Most recent overnight admission** | **Most recent observation room/ emergency room visit** | **Most recent routine outpatient visit** |
| **2.6** | How did you get to the hospital? | 1 = walk  2 = bicycle  3 = scooter or motorcycle  4 = public bus or van  5 = private car  6 = taxi  7 = ambulance  66 = Other  88 = Don’t know  99 = Refused |  |  |  |
| **2.6** | How many people went with you on this trip?  ***ENTER NO OF PEOPLE*** | 00 = Patient went alone  88 = Don’t know  99 = Refused |  |  |  |
| **2.6** | How much did it cost to travel to the hospital?  ***ENTER ONE-WAY AMOUNT FOR ALL PERSONS WHO TRAVELLED*** | 88 = Don’t know  99 = Refused |  |  |  |
| **2.6** | How much did you and your family pay for unofficial costs that were not issued a receipt during this hospital visit?  ***ENTER AMOUNT*** | 88 = Don’t know  99 = Refused |  |  |  |
| **2.6** | How much did you and your family pay for everything that happened at this hospital visit including the cost of medicine, tests done on you, food, fees paid to doctors and laboratory costs, but excluding travel costs?  ***ENTER AMOUNT*** | 88 = Don’t know  99 = Refused |  |  |  |

| **Question** | | **Codes** | **Response** |
| --- | --- | --- | --- |
| **3.2 ** | Now I want to ask you about health care you received at places other than a hospital. Again, I only want to know about care you received within the last 90 days since [mention anchor event]. During the last 90 days, did you seek health care services from places other than a hospital? | 1 = Yes  ***GO TO SECTION 3***  2 = No  ***SKIP TO SECTION 3***  88 = Don’t know  ***SKIP TO SECTION 3***  99 = Refused  ***SKIP TO SECTION 3***  ***IF NO, MAKE SURE NO HOSPITAL VISIT OF ANY KIND*** |  |

| **Non hospital visits** | **Questions with codes** | | | |
| --- | --- | --- | --- | --- |
|  | **3.2**  How many visits did you make to the [*name each provider in turn from column on left*] in the last 90 days? | **3.2**  How long did it take you to travel one way on your most recent visit to the [*name each provider in turn from column on left*]?  ***ENTER AS A DECIMAL I.E. 1H30MINS = 1.5HOURS*** | **3.2**  What was the total cost of travelling one way on your most recent visit to the [*name each provider in turn from column on left*]?  ***ENTER ONE-WAY AMOUNT FOR ALL PERSONS WHO TRAVELLED*** | **3.2**  How much did you and your family pay for everything that happened during your last visit to the [*name each provider in turn from column on left*] including the cost of medicine, tests done on you, food, fees paid to doctors and laboratory costs, but excluding travel costs?  ***ENTER AMOUNT*** |
| **Specialist Doctor or Surgeon** |  |  |  |  |
| **Primary Care Doctor** |  |  |  |  |
| **Nurse** |  |  |  |  |
| **Pharmacist or Dispensary** |  |  |  |  |
| **Visit to a clinic to collect medication** |  |  |  |  |
| **Health Educator** |  |  |  |  |
| **Community Health Worker** |  |  |  |  |

| **Question** | | **Health Conditions** | **Response** |
| --- | --- | --- | --- |
| **2.1** | I will now read you a list of health conditions that people sometimes have. Please tell me which ones a doctor has EVER said you have.  ***ENTER CODES:***  **1 = Yes**  **2 = No**  **88 = Don’t know**  **99 = Refused** | Heart attack, heart failure or other heart disease |  |
|  |  | Stroke |  |
|  |  | High blood pressure |  |
|  |  | Cancer |  |
|  |  | Asthma |  |
|  |  | Other lung disease |  |
|  |  | HIV/AIDS |  |
|  |  | Erectile dysfunction or loss of libido |  |
|  |  | Kidney disease |  |
|  |  | Amputation of toe, foot, or leg |  |
|  |  | Eye surgery |  |
|  |  | Peripheral neuropathy |  |
|  |  | Foot or leg ulcer |  |
|  |  | Laser treatment on your eyes |  |
|  |  | Kidney Dialysis |  |
|  |  | Depression |  |
|  |  | Other mental condition |  |
|  |  | Diabetes (type 1) |  |
|  |  | Diabetes (type 2) |  |
|  |  | Other permanent problem you still have *(specify)*  …………………………………………………. |  |
| **2.1** | Now I want to ask about temporary health problems that you have had. Within the last 90 days, since [*mention anchor event*], have you had…  ***ENTER CODES:***  **1 = Yes**  **2 = No**  **88 = Don’t know**  **99 = Refused** | Active tuberculosis |  |
|  |  | Influenza or Flu |  |
|  |  | Pneumonia |  |
|  |  | Diarrhoea lasting 3 or more days or requiring drip or pills |  |
|  |  | Pregnancy or childbirth |  |
|  |  | Other major infection |  |
|  |  | Injury |  |
|  |  | ‘Cold’ or other virus |  |
|  |  | Typhoid |  |
|  |  | Cholera |  |
|  |  | Other temporary illness *(specify)*  …………………………………………………. |  |
| **2.1** | During the last 90 days (3 months), did someone perform any of these kinds of tests on you, including in a hospital? Please do not count tests you did yourself.  ***ENTER CODES:***  **1 = Yes**  **2 = No**  **88 = Don’t know**  **99 = Refused** | Any urine test |  |
|  |  | Test on your blood taken by needle |  |
|  |  | Finger-stick blood test |  |
|  |  | Blood pressure measurement (cuff on arm) |  |
|  |  | Eye exam |  |
|  |  | A test of your feet for feeling |  |
|  |  | Taking your weight on a scale |  |
|  |  | Measuring your waist with a tape |  |
|  |  | Sputum test |  |
|  |  | Chest x-ray |  |
|  |  | Other test *(specify)*  …………………………………………………. |  |

1. **IMPACT OF HEALTH PROBLEMS**

| **Question** | | **Codes** | **Responses** | | | |
| --- | --- | --- | --- | --- | --- | --- |
|  |  |  | **Respondent** | **Family member** | **Family member** | **Family member** |
| **2.6** | Now I would like to ask you about your health problems are affecting your life and the life of your family. Are your health problems preventing you from doing any paying work? | ***CODES FOR QUESTIONS ABOUT RESPONDENT:***  1 = Yes  2 = No  88 = Does not apply/ Don’t know  99 = Refused  ***CODES FOR QUESTIONS ABOUT FAMILY MEMBERS:***  1 = Mother  2 = Father  3 = Brother  4 = Sister  5 = Daughter  6 = Son  7 = Granddaughter  8 = Grandson  10 = Husband  11 = Wife  66 = Other  88 = Does not apply/ Don’t know  99 = Refused  ***IN THIS TABLE, FAMILY IS NOT LIMITED TO PERSON WITH WHO THE RESPONDENT EATS BUT ALSO INCLUDES ANYONE RELATED BY BLOOD OR ADOPTION*** |  |  |  |  |
| **2.6** | Are your health problems keeping you from doing as much paying work as you would like? |  |  |  |  |  |
| **2.6** | Are your health problems making you do more paying work than you otherwise would? |  |  |  |  |  |
| **2.6** | Are your health problems preventing some other person in your family from doing any paying work? |  |  |  |  |  |
| **2.6** | Are your health problems preventing some other person in your family from doing any paying work? |  |  |  |  |  |
| **2.6** | Are your health problems keeping some other person in your family from doing as much paying work as they would like? |  |  |  |  |  |
| **2.6** | Are your health problems making other family members do more paying work than they would like? |  |  |  |  |  |
| **2.6** | Are your health problems preventing you from growing any food or doing any work in the house? |  |  |  |  |  |
| **2.6** | Are your health problems preventing some other person in your family from growing any food or doing any work in the house? |  |  |  |  |  |
| **2.6** | Are your health problems keeping you from doing as much farming or housework as you would like? |  |  |  |  |  |
| **2.6** | Are your health problems preventing some other person in your family from growing as much food or doing as much work in the house? |  |  |  |  |  |
| **2.6** | Are your health problems preventing you from enrolling in school or training? |  |  |  |  |  |
| **2.6** | Are your health problems keeping you from seeing friends or family? |  |  |  |  |  |
| **2.6** | Are your health problems keeping you from getting enough to eat? |  |  |  |  |  |
| **2.6** | Are your health problems preventing any other member of your family from getting enough to eat? |  |  |  |  |  |

| **Question** | | **Codes** | **Response** |
| --- | --- | --- | --- |
| **1.3** | During the last 90 days, how many days did you miss your usual activities (work, work at home or school) because of health reasons?  ***FILL IN THE NUMBER OF DAYS.*** | 88 = Does not apply/ Don’t know  99 = Refused |  |
| **1.4** | Have you or your family hired someone to take care of your because of your ill health? | 1 = Yes  2 = No  ***SKIP TO QUESTION 3***  88 = Don’t know  99 = Refused |  |
| **1.4** | How much do you pay this person each month?  ***FILL IN THE AMOUNT*** | 1 = Yes  2 = No  88 = Don’t know  99 = Refused |  |
| **1.4** | Does someone from your family who is not paid, take care of your because of your ill health? | 1 = Yes  2 = No  ***SKIP TO QUESTION 3***  88 = Don’t know  99 = Refused |  |
| **1.4** | How much of this person’s day is spent taking care of you? | 1 = 1-25%  2 = 26-50%  3 = 51-75%  4 = 76-100%  88 = Don’t know  99 = Refused |  |
| **2.1** | During the past 12 months, how did you pay for or get the medical services, caregivers, medicines and medical supplies that you, yourself, used?  ***ENTER CODES:***  **1 = Yes**  **2 = No**  **88 = Don’t know**  **99 = Refused** | By spending out of your current HOUSEHOLD income |  |
|  |  | Social welfare support |  |
|  |  | Donations of medicines or supplies by employers or agencies |  |
|  |  | With money or fundraising from friends or family who live with you or near you |  |
|  |  | With money or fundraising from family living abroad |  |
|  |  | From saved money, such as bank account savings |  |
|  |  | By borrowing money |  |
|  |  | By selling possessions like furniture, animals or jewellery |  |
|  |  | By selling housing or land |  |
|  |  | Other *(specify)*  …………………………………………………. |  |
| **2.1** | Now I am going to read you a list of reasons why people sometimes do not get medical services when they want them. Please tell me which if any of these have prevented you from getting medicines or medical care, over the last 12 months?  ***ENTER CODES:***  **1 = Yes**  **2 = No**  **88 = Don’t know**  **99 = Refused** | Lack of funds to pay for the care |  |
|  |  | Lack of transportation |  |
|  |  | No one to help me get there |  |
|  |  | Would have taken to long to go there/ too far away |  |
|  |  | Would have had to wait too long once I was there |  |
|  |  | Did not have health insurance |  |
|  |  | Too sick to make the trip |  |
|  |  | No doctor or other professional was available |  |
|  |  | Medicines were not in stock |  |
|  |  | Did not trust the care |  |
|  |  | Did not know where to go |  |
|  |  | Some other reason *(specify)*  …………………………………………………. |  |

1. **MEDICATIONS**

| **Question** | | **Codes** | **Response** |
| --- | --- | --- | --- |
| **1.4** | Are you currently taking any other pills or injections? | 1 = Yes  2 = No  ***SKIP TO SECTION 3***  88 = Don’t know  99 = Refused |  |
| **1.4** | If YES, may I see the pills and injections you use and ask a few questions about them? If there is any medication that you do not have with you today could you describe it to me?  ***HAVE THE RESPONDENT SHOW YOU ALL THE PILLS, INSULIN, AND OTHER MEDICINES THAT HE OR SHE IS CURRENTLY TAKING. IF THE RESPONDENT DOES NOT HAVE THEM TO HAND, PLEASE ASK THEM TO GIVE YOU THE DETAILS AND WRITE A 2 IN THE LAST COLUMN.*** | | |

| **Question** | **5.3**  **Compound (medicine), or type of insulin, or type of insulin syringe or pen (if not pre-filled insulin)** | **5.4**  **Mg per pill or insulin units taken per day** | **5.5**  **Number of pills or shots prescribed per day** | **5.6**  **Number of pills/shots taken per day (when taken)** | **5.7**  **If not taken as prescribed, why not (primary reason)** | **5.8**  **Where was the medication obtained** | **5.9**  **How much did you pay the last time you bought this medicine?** | **5.10**  **How many pills or units of insulin did you get?** | **5.11**  **How many doses of short course chemotherapy did you get?** |
| --- | --- | --- | --- | --- | --- | --- | --- | --- | --- |
| **Codes and Instructions** |  |  |  |  | 0 = as prescribed  1 = side effects  2 = cannot afford  3 = forget  4 = hard to get  5 = help not available  6 = syringe equipment problems  7 = don’t believe it works  8 = don’t need it  88 = don’t know  99 = refused | 1 = private pharmacy  2 = hospital pharmacy  3 = clinic  4 = relative or friend  66 = other  88 don’t know  99 = refused | If cost of medicine was part of doctor or hospital fee enter 000 | If not diabetic enter 000 | If not affected by tuberculosis enter 000 |
| **List of medications** |  |  |  |  |  |  |  |  |  |
|  |  |  |  |  |  |  |  |  |
|  |  |  |  |  |  |  |  |  |
|  |  |  |  |  |  |  |  |  |
|  |  |  |  |  |  |  |  |  |
|  |  |  |  |  |  |  |  |  |
|  |  |  |  |  |  |  |  |  |
|  |  |  |  |  |  |  |  |  |
|  |  |  |  |  |  |  |  |  |

1. **QUESTIONS FOR PATIENTS WITH DIABETES**

| **Question** | | **Codes** | **Response** |
| --- | --- | --- | --- |
| **6.1** | For how many years have you had diabetes? | ***FILL IN THE NUMBER OF YEARS AND MONTHS.***  88 = Don’t know  99 = Refused | **…………yrs**  **………..mths** |
|  | Do you recall when your symptoms first began? | 1 = Yes  2 = No  ***SKIP TO QUESTION 3***  88 = Don’t know  99 = Refused |  |
|  | Do you recall when you were first diagnosed? | 1 = Yes  2 = No  ***SKIP TO QUESTION 3***  88 = Don’t know  99 = Refused |  |
|  | Approximately how long did it take for you to get an accurate diagnosis from when your symptoms first began? | ***FILL IN THE NUMBER OF YEARS, MONTHS OR DAYS.*** | **…………yrs**  **………..mths**  **………..days** |
|  | Approximately how long did it take for you to begin receiving appropriate treatment, from when you first received a diagnosis? | ***FILL IN THE NUMBER OF YEARS, MONTHS OR DAYS.*** | **…………yrs**  **………..mths**  **………..days** |
|  | During the last 90 days did you test your own blood sugar? | 1 = Yes  2 = No  ***SKIP TO QUESTION 3***  88 = Don’t know  99 = Refused |  |
|  | During the last 90 days how often did you test your own blood sugar? | 1 = More than once a day  2 = Twice a day  3 = Once a day  4 = Once or twice a week  5 = Less than once a week  6 = Once a months  7 = Once in three months  88 = Don’t know  99 = Refused |  |
|  | How many blood sugar testing strips did you receive, the last time you got some | ***FILL IN THE NUMBER***  888 = Don’t know  999 = Refused |  |
|  | Where did you get these testing strips | 1 = Private Doctor  2 = Private Pharmacy  3 = Sent from abroad by family  4 = Donation  5 = Given to me at a public clinic  6 = Other ………………………….***SPECIFY***  88 = Don’t know  99 = Refused |  |
|  | Over the last year, have you been able to get all the testing strips you need, when you needed them? | 1 = Yes, always  2 = Most of the time  3 = Usually not  88 = Don’t know  99 = Refused |  |
|  | During the last 90 days, how often has someone else measured your blood sugar, not counting tests your family did for you? | 1 = More than twice a day  2 = Twice a day  3 = Once a day  4 = Once or twice a week  5 = Less than once a week  6 = Once a months  7 = Once in three months  88 = Don’t know  99 = Refused |  |
|  | When the cost of blood sugar test is separate from hospitals, clinics or doctors visit costs, how much do you usually pay to have someone perform one blood sugar test? | ***FILL IN THE AMOUNT***  888 = Don’t know  999 = Refused |  |
|  | Have you injected insulin at home or in a nearby clinic during the last 90 days? | 1 = Yes, I inject myself at home  2 = Yes, a family member injects me at home  3 = Yes, a paid carer injects me at home  4 = Yes, I get my injection at a public clinic  5 = No  88 = Don’t know  99 = Refused |  |
|  | What are your main reasons for not using insulin? | 1 = Insulin not prescribed by my doctor  2 = Insulin too expensive – I cannot afford to buy it  3 = Insulin not available/in stock in pharmacy or clinic  4 = Insulin too weak, too strong, or adulterated (poor quality)  5 = Syringes not in stock in pharmacy or clinic near my home  6 = Syringes too expensive – I cannot afford to buy them  7 = Afraid of or dislike needles  8 = Don’t know how to inject insulin properly  9 = Don’t know when to inject insulin  10 = Doctor prescribed it but I don’t think I need it  11 = Cannot find or afford blood sugar testing supplies  12 = Doctor or nurse not available in the clinic  66 = Other reason  88 = Don’t know  99 = Refused |  |
|  | During the last 90 days, on how many days did you fail to inject your insulin because you did not have useable syringes or needles? | 1 = Never  2 = 5 days or fewer  3 = 6 – 10 days  4 = 11 – 20 days  5 = 21 – 45 days  6 = More than half the time  7 = Always (did not inject insulin in last 90 days because did not have syringes)  88 = Don’t know  99 = Refused |  |
| **1.4** | During the last 90 days, about how many times did you reuse each needle, to give yourself insulin? | 1 = Never (I always used a needle for each injection)  2 = Twice  3 = Three times  4 = Four times  5 = Five times or more  88 = Don’t know  99 = Refused |  |

1. **QUESTIONS FOR PATIENTS WITH TUBERCULOSIS**

| **Question** | | **Codes** | **Response** |
| --- | --- | --- | --- |
|  | Is this the first time you have had TB? | 1 = Yes  2 = No  88 = Don’t know  99 = Refused |  |
| **6.1** | For how long have you had TB? | ***FILL IN THE NUMBER OF YEARS AND MONTHS. REFER ONLY TO THE CURRENT INFECTION.***  88 = Don’t know  99 = Refused | **…………wks**  **………mths** |
|  | Do you recall when your symptoms first began? | 1 = Yes  2 = No  ***SKIP TO QUESTION 3***  88 = Don’t know  99 = Refused |  |
|  | Do you recall when you were first diagnosed? | 1 = Yes  2 = No  ***SKIP TO QUESTION 3***  88 = Don’t know  99 = Refused |  |
|  | Approximately how long did it take for you to get an accurate diagnosis from when your symptoms first began? | ***FILL IN THE NUMBER OF YEARS, MONTHS OR DAYS.*** | **…………yrs**  **………..mths**  **………..days** |
|  | Approximately how long did it take for you to begin receiving appropriate treatment, from when you first received a diagnosis? | ***FILL IN THE NUMBER OF YEARS, MONTHS OR DAYS.*** | **…………yrs**  **………..mths**  **………..days** |
|  | On average, how often do you take your TB medication? | 1 = More than twice a day  2 = Twice a day  3 = Once a day  4 = Once or twice a week  5 = Less than once a week  6 = Once a months  7 = Once in three months  8 = Do not take medication  ***SKIP TO QUESTION 3***  88 = Don’t know  99 = Refused |  |
|  | Over the course of your treatment, have you been able to get all the medication you need, when you needed it? | 1 = Yes, always  2 = Most of the time  3 = Usually not  88 = Don’t know  99 = Refused |  |
|  | When the cost of TB medication is separate from hospitals, clinics or doctors visit costs, how much do you usually pay for your medication? | ***FILL IN THE AMOUNT***  888 = Don’t know  999 = Refused |  |
|  | During the last 90 days, on how many days did you fail to take your TB medication as it was prescribed?? | 1 = Never  ***SKIP TO QUESTION 3***  2 = 5 days or fewer  3 = 6 – 10 days  4 = 11 – 20 days  5 = 21 – 45 days  6 = More than half the time  7 = Always (did not inject insulin in last 90 days because did not have syringes)  88 = Don’t know  99 = Refused |  |
|  | What are your main reasons for not taking your TB medication? | 1 = Medication not prescribed by my doctor  2 = Medication too expensive – I cannot afford to buy it  3 = Medication not available/in stock in pharmacy or clinic  4 = Medication too weak, too strong, or adulterated (poor quality)  5 = The side effects are too unpleasant  6 = Afraid of or dislike the medication  7 = Don’t know how to take the medication  8 = Don’t know when to take the medication  9 = Doctor prescribed it but I don’t think I need it  10 = I feel I have recovered no and no longer need the medication  11 = Doctor or nurse not available in the clinic  66 = Other reason  88 = Don’t know  99 = Refused |  |

1. **STIGMA**

| **Q.N.** | **Questions** | **Coding** | **Code** | **Skip** |
| --- | --- | --- | --- | --- |
| 901 | I would now like to ask you about the times when you have been excluded from various activities in the past year on account of your disability. In the past 12 months how often have you......  Been excluded from social gatherings or activities (e.g. weddings, funerals, parties, clubs)?  Been excluded from religious activities or places of worship?  Been excluded from family activities (e.g. cooking, eating together, sleeping in the same room?)  Been aware of being gossiped about you?  Been forced to change your place of residence or been unable to rent accommodation?  Lost a job (if employed) or another source of income (if self-employed or an informal/casual worker)?  Been excluded/isolated in work place  Been dismissed, suspended or prevented from attending an educational institution  Has your child/children been dismissed, suspended or prevented from attending an educational institution?  Been denied health services? | Never Once A few times Often  1 2 3 4    1 2 3 4    1 2 3 4    1 2 3 4  1 2 3 4    1 2 3 4    1 2 3 4      1 2 3 4  1 2 3 4 |  |  |
| 902 | In the last 12 months, have you experienced any of the following feelings on account of your disability? | Yes No |  |  |
|  | I feel ashamed | 1 2 |  |  |
|  | I feel guilty | 1 2 |  |  |
|  | I blame myself | 1 2 |  |  |
|  | I blame others | 1 2 |  |  |
|  | I feel very helpless | 1 2 |  |  |
|  | I feel I should be punished | 1 2 |  |  |
|  | I feel suicidal | 1 2 |  |  |
| 903 | In the last 12 months, have you done any of the following things? | **Yes No** |  |  |
|  | I have chosen not to attend social gathering(s) | 1 2 |  |  |
|  | I have isolated myself from my family and/or friends | 1 2 |  |  |
|  | I took the decision to stop working | 1 2 |  |  |
|  | I decided not to apply for a job/work or for a promotion | 1 2 |  |  |
|  | I withdrew from education/training or did not take up an opportunity for education/training | 1 2 |  |  |
|  | I decided not to get married | 1 2 |  |  |
|  | I decided not to have sex | 1 2 |  |  |
|  | I decided not to have (more) children | 1 2 |  |  |
|  | I avoided going to a local clinic when I needed to | 1 2 |  |  |
|  | I avoided going to a hospital when I needed to | 1 2 |  |  |

1. Survey IDs should be unique and should also tell us where the interview was conducted for example interview conducted in the diabetes clinic should start with 10 and in the TB clinic with 20, followed then by the number of the interview in each clinic 1001,1002,1003 etc and 2001, 2002 etc. [↑](#footnote-ref-2)
